# Beyond the hospital door: a retrospective, cohort study of associations between birthing in the public or private sector and women’s postpartum care

**DOI:** 10.1186/s12913-015-0689-3

**Published:** 2015-01-22

**Authors:** Wendy Brodribb, Maria Zadoroznyj, Michelle Nesic, Sue Kruske, Yvette D Miller

**Affiliations:** Discipline of General Practice, School of Medicine, The University of Queensland, Royal Brisbane and Women’s Hospital, Level 8, Health Sciences Building, Herston, QLD 4029 Australia; Institute for Social Science Research, School of Social Science, The University of Queensland, 4th floor, GPN3 (Building 39A), St Lucia, QLD 4072 Australia; Queensland Centre for Mothers & Babies, School of Psychology, The University of Queensland, Hood St, St Lucia, QLD 4072 Australia; School of Nursing and Midwifery, The University of Queensland, Level 2, Edith Cavell Building, UQ Herston Campus, Herston, QLD 4029 Australia; School of Public Health and Social Work, Queensland University of Technology, Victoria Park Rd, Kelvin Grove, QLD 4059 Australia

**Keywords:** Postnatal, Community, Maternal satisfaction, Health insurance, Parenting confidence, Postnatal depression

## Abstract

**Background:**

In Australia, maternity care is available through universal coverage and a parallel, competitive private health insurance system. Differences between sectors in antenatal and intrapartum care and associated outcomes are well documented but few studies have investigated differences in postpartum care following hospital discharge and their impact on maternal satisfaction and confidence.

**Methods:**

Women who birthed in Queensland, Australia from February to May 2010 were mailed a self-report survey 4 months postpartum. Regression analysis was used to determine associations between sector of birth and postpartum care, and whether postpartum care experiences explained sector differences in postpartum well-being (satisfaction, parenting confidence and feeling depressed).

**Results:**

Women who birthed in the public sector had higher odds of health professional contact in the first 10 days post-discharge and satisfaction with the amount of postpartum care. After adjusting for demographic and postpartum contact variables, sector of birth no longer had an impact on satisfaction (AOR 0.95, 99% CI 0.78-1.31), but any form of health professional contact did. Women who had a care provider’s 24 hour contact details had higher odds of being satisfied (AOR 3.64, 95% CI 3.00-4.42) and confident (AOR 1.34, 95% CI 1.08- 1.65).

**Conclusion:**

Women who birthed in the public sector appeared more satisfied because they had higher odds of receiving contact from a health professional within 10 days post-discharge. All women should have an opportunity to speak to and/or see a doctor, midwife or nurse in the first 10 days at home, and the details of a person they can contact 24 hours a day.

## Background

Like many other high-income welfare states such as Canada, the UK and the Netherlands, maternity care in Australia is available through a system of universal health coverage available to all citizens [1]. However, Australia is distinctive in its parallel private health insurance system which operates alongside and in competition with the public sector [1-5]. Approximately 71% of women use the public sector [6] where they receive either midwifery or medically led care. The remaining 29% choose to birth in private facilities under the care of a private obstetrician.

In recent years, a range of government policies and direct subsidies to insurers have increased the proportion of women whose maternity care is provided within the private sector [2-4,7] from 25.4% in 2000 [8] to 29.0% in 2011 [6]. These policies have been criticised as costly and inequitable, favouring the most affluent segments of the Australian population and those in metropolitan areas [3,9-12]. They have also stratified maternal and neonatal outcomes in Australia [6,7,13,14]. Compared with women who birth in public facilities, women who birth in private facilities have higher rates of instrumental delivery, caesarean birth, induction of labour, episiotomy and epidural analgesia [7,15-18].

Length of hospital stay after birth also varies by sector. The median length of stay is 4 days in the private sector and 2 days in the public sector [6]. However, the substantial literature on the effects of sector on maternity care has tended to stop at the hospital door, with little research into the implications of sector on women’s experiences following discharge [14]. One study found a higher prevalence of depressive symptoms at 6–8 weeks postpartum among women who had birthed in public hospitals, although this association may be confounded by lower income and educational levels [19].

Australian women report high levels of dissatisfaction, unmet needs and confusion about where to get help as they transition to motherhood [20-24]. New mothers, especially first-time mothers, also report a lack confidence in their ability to care for their baby [25].

In Australia, post-birth care in the community is shaped by both sector and geographic location. While the Australian Government provides much of the funding for health care and determines national policy, the six State and two Territory governments have responsibility for the provision and ongoing management of health services within their jurisdictions [26]. Community based post-birth care may be provided by caseload midwives [27] or domiciliary midwives employed by birthing facilities, by private midwifery services, by general practitioners (GPs), and/or by child and family health nursing services (CFHNs) routinely available to all pregnant women, new mothers, children, and their families [28]. The reach of these services and the level of coordination between them varies greatly across and within jurisdictions. Specialist obstetric and paediatric services are rarely accessed before six weeks postpartum, and then only by some women who birth in the private sector.

In the State of Queensland, although publically funded, universal postpartum care is available through CFHNs, access varies greatly [22,29]. In 2008, the Queensland Health department established the Universal Postnatal Contact Services program (UPNCS). This program aimed to provide universal follow-up of new mothers shortly after hospital discharge (either by telephone or home visiting) and improve access to community-based, drop-in centres. However, private health services were not included and public services implemented the program inconsistently across the State [30].

To date there is limited published research on how postpartum care in the community (after discharge from hospital) is provided to Australian women and if their experiences vary across sectors. A qualitative study of Queensland women suggests that women who birth in the private sector are less likely to be given information about community follow-up and often feel abandoned and confused on leaving hospital [31]. However, it is unknown if these findings translate to poorer outcomes for women in the private sector.

Our study aimed to determine: 1. associations between sector of birth facility and community postpartum care, 2. associations between sector of birth facility and satisfaction with postpartum care, parenting confidence, and feeling depressed after birth, and 3. whether differences in postpartum care explain any differences found in satisfaction with care or in parenting confidence between women who gave birth in public and private sector facilities in Queensland.

## Methods

### Participants and procedure

Participants were women who completed the *Having a Baby in Queensland 2010 Survey*, a cross-sectional retrospective self-report survey of women birthing in Queensland, Australia. All women who: 1. had a live single or multiple birth in Queensland from 1st February to 31st May 2010; 2. did not have a neonatal death; and, 3. had an accurate mailing address in their Queensland Registry of Births, Deaths and Marriages records, were mailed a survey approximately 4 months after birth. Women could complete the paper survey and return it by reply paid mail, complete the survey over the telephone (using a translator if required) or via a secure online survey system. Reminder/thank you slips were mailed two weeks after the initial survey. Further details about the survey procedure can be found elsewhere [24]. Women who gave birth at home were excluded from this analysis.

Ethical approval for the *Having a Baby in Queensland 2010 Survey* was obtained from the Behavioural and Social Sciences Ethical Review Committee of The University of Queensland.

### Measures

The measures uses in this analysis are found in Table 1. A complete copy of the survey can be found at http://www.qcmb.org.au/overview_of_the_survey_program#1.

**Table 1:** **Summary of variables included in the analysis**

**Variable**	**Measure**	**Categories**	**Coding**
Sector of birth facility	Did you have your baby in a private or public facility?	1. Public hospital or public birth centre	Missing data was imputed by using other responses pertaining to their birth hospital and public or private status.
		2. Private hospital or private birth centre	
		3. Don’t know	
Postpartum care	In the first 10 days of being at home with your baby, did any of the following happen?	1. I was telephoned by a midwife or nurse	Women could nominate multiple contacts. Responses 1 and 2 were deemed to have been *proactively delivered by the health service.*
		2. I was visited at home by a midwife or nurse	Responses 3 and 4 were deemed to have been *initiated by the women*.
		3. I visited a midwife or nurse	Women whose babies had not come home were not included in the analysis.
		4. I visited a general practitioner (GP)	
		5. None of the above	
		6. My baby hasn’t come home yet	
Access to 24-hour support	When you were at home after the birth of your baby, did you have the name and contact details of a care provider you could get in touch with at any hour if you were worried?	1. Yes	
		2. No	
Length of hospital stay	Altogether, how long did you stay in the hospital or birth centre where your baby was born?	1. Nights	The variable was transformed into a categorical variable consisting of four groups: *<24 hours, 1–2 nights, 3–4 nights, 5+ nights*. Women whose babies were born at home were not included in the analysis.
		2. Hours	
		3. I had my baby at home	
Satisfaction with the amount of postnatal care	Thinking about the amount of contact you had with care providers since having your baby, in your opinion, was this…	1. Too much?	‘About right’ was coded as satisfied with amount of postnatal care and ‘too much’ and ‘too little’ was coded as not satisfied with amount of postnatal care.
		2. Too little?	
		3. About right?	
Parenting confidence	When you first had your new baby at home, how confident did you feel about looking after him or her?	1. Extremely confident	‘Extremely confident’ was coded as *confident*, and ‘fairly confident’ to ‘not at all confident’ was coded as *not confident*. This categorisation was chosen to reflect an optimal outcome in parenting confidence.
		2. Fairly confident	
		3. Confident	
		4. Not very confident	
		5. Not at all confident	
		6. My baby hasn’t come home yet	
Feeling depressed	How often have you experienced feeling depressed since having your baby?	1. Never	Women were coded as having experienced *feeling depressed* since their birth if they responded ‘sometimes’ or ‘often’
		2. Rarely	
		3. Sometimes	
		4. Often	
		5. Does not apply to me	
Maternal age at time of birth of the baby	What was your date of birth?		Age was derived from maternal date of birth and date of birth of the baby and coded as *<20, 20–24, 25–29, 30–34, 35–39, ≥40*
	When was your baby born?		
Identification as an Aboriginal and/or Torres Strait Islander person	Which of the following best describes you?	1. Aboriginal	The variable was coded as *ATSI culture* yes, *no.*
		2. Torres Strait Islander	
		3. South Sea Islander	
		4. None of the above	
Country of birth	Where were you born?	1. Australia	
		2. Other country	
Level of education	What is the highest level of qualification you have completed?	1. No formal qualifications	The variable was transformed into four categories: *Year 10* (responses 1 and 2), *Year 12* (response 3), *Trade/diploma* (responses 4 and 5) and *university* (responses 6 and 7)
		2. Year 10 or equivalent	
		3. Year 12 or equivalent	
		4. Trade/apprenticeship	
		5. Certificate/diploma	
		6. University degree	
		7. Postgraduate degree	
Area of residence	In what town or suburb was your usual place of residence you’re your baby was born?		Area of residence was calculated from postcode using Accessibility/Remoteness Index of Australia and classified as *City, inner regional, outer regional* and *remote*.
	What is the postcode of this town or suburb?		

### Data analysis

Data were analysed using IBM SPSS Statistics for Windows (version 21.0) [32]. Chi square analyses were used to examine differences between the sample and the population of birthing women in Queensland to assess sample representativeness. All further analyses were adjusted for non-modifiable demographic variables known to affect access to care (age, parity, level of education, country of birth, Aboriginal and/or Torres Strait Islander identification and area of residence) to remove any variance associated with them. A series of multivariate logistic regression analyses were conducted to examine associations between sector of birth and postpartum care including length of hospital stay. Hierarchical multivariate binary logistic regression analysis was used to assess factors associated with satisfaction with the amount of postpartum contact with care providers, parenting confidence and feeling depressed in the time after birth. We chose to use logistic regression with dichotomous coding of the ‘top score’ for all outcomes (rather than assessing associations with a more graded response) to enable the prediction of markers for optimal care.

Demographic variables were simultaneously entered at Step 1. Birthing sector was entered at Step 2. To determine associations between initiatives proactively delivered by health services and each outcome, length of hospital stay, being telephoned by a care provider, visited at home by a care provider and given contact details of someone to contact any time, were simultaneously entered at Step 3. The final step in the model contained additional initiatives undertaken by women (having visited a midwife or nurse and having visited a GP).

Due to the large sample size, Alpha was set at 0.01 for all analyses.

## Results

### Sample characteristics

After exclusions (due to neonatal death and lack of contact details (N = 99) and returned to sender (N = 543)), 20 371 survey packages were delivered. Of the 7,193 (35.3%) responses, 760 were excluded from this analysis because the woman birthed outside a hospital or birth centre or was missing data for sector of birth facility, postpartum care, satisfaction with postpartum care, parenting confidence, feeling depressed after birth, or for a covariate, leaving 6433 women for inclusion.

The majority of women were multiparous (54.4%), had completed secondary school (90.3%), resided in a city (64.0%), were born in Australia (80.4%) and did not identify as Aboriginal or Torres Strait Islander (98.4%) (see Table 2).

**Table 2:** **Sample characteristics by sector of birth and compared to the total population of birthing women**

	**Public facility**	**Private facility**	**Whole sample**	**Population of birthing women**	**Chi-square (df)** ***p*** ^**1**^
	**% (N)**	**% (N)**	**% (N)**	**% (N)** [33]	
**Parity**					
*Primiparous*	45.2 (1696)	46.2 (1239)	45.6 (2935)	40.8 (24,878)	*χ* ^*2*^(1) = 62.68
*Multiparous*	54.8 (2055)	53.8 (1443)	54.4 (3498)	59.2 (36,149)	*p* < .001
**Area of residence**					
*City*	58.8 (2204)	71.3 (1911)	64.0 (4115)	59.7(36,452)	*χ* ^*2*^(3) = 57.03*,*
*Inner Regional*	22.2 (833)	15.6 (418)	19.4 (1251)	20.7 (12,661)	*p* < .001
*Outer Regional*	16.1 (604)	11.5 (308)	14.2 (912)	16.2 (9,980)	
*Remote*	2.9 (110)	1.7 (45)	2.4 (155)	3.3 (1980)	
**Age**					
*<20 years*	3.5 (133)	0.2 (6)	2.2 (139)	5.5 (3,344)	*χ* ^*2*^(5) = 660.72
*20-24*	16.6 (622)	4.3 (114)	11.4 (736)	17.4 (10,616)	*p* < .001
*25-29*	33.0 (1239)	23.8 (638)	29.2 (1877)	28.4 (17,314)	
*30-34*	28.3 (1060)	42.1 (1129)	34.0 (2189)	28.9 (17,607)	
*35-39*	15.2 (571)	25.1 (674)	19.4 (1245)	16.5 (10,037)	
*>40*	3.4 (126)	4.5 (121)	3.8(247)	3.5 (2,109)	
**Aboriginal and/or Torres Strait Islander**					
*No*	97.4 (3654)	99.7 (2674)	98.4 (6328)	94.2 (57,511)	*χ* ^*2*^(1) = 200.78
*Yes*	2.6 (97)	0.3(8)	1.6 (105)	5.8 (3,511)	*p* < .001
**Country of Birth**					
*Australia*	77.3 (2898)	84.9 (2276)	80.4 (5174)	77.3 (47,191)	*χ* ^*2*^ *(1) = 34.27*
*Outside Australia*	22.7 (853)	15.1 (406)	19.6 (1259)	22.7 (13,798)	*p* < .001
**Level of Education**					
*Year 10*	14.2 (531)	3.4 (90)	9.7 (621)		
*Year 12*	23.2 (869)	13.8 (370)	19.3 (1239)		
*Trade/Diploma*	33.8 (1269)	23.9 (641)	29.7 (1910)		
*University*	28.8 (1082)	58.9 (1581)	41.4 (2663)		

^1^Difference between sample and population.

Compared with the population of birthing women in Queensland in 2010 [33] the sample somewhat under-represented women who were younger than 20 years of age (2.2% in the study sample vs. 5.5% in the Queensland population), were multiparous (54.4% vs. 59.2%) identified as Aboriginal or Torres Strait Islander (1.6% vs. 5.8%) and over represented women living in a city (64.0% vs 59.7%) (see Table 2). Women who birthed in a public facility were also under-represented in the sample (58.3% vs. 69.0% *χ*^2^(1) = 344.77 *p* < .001).

### Associations between sector of birth facility and postpartum care

After adjustment for demographic factors, women who birthed in a public facility were more likely than women who birthed in a private facility of staying in hospital less than 24 hours after birth, and of receiving the details of a care provider they could contact at any time (see Table 3). They also had more than six times higher odds of being telephoned by a care provider, more than 34 times the odds of being visited at home, and more than five times the odds of visiting a GP within 10 days of being at home than women who birthed in a private facility (see Table 3). Women birthing in a public facility had significantly lower odds of visiting a nurse or midwife in the 10 days after their hospital discharge (see Table 3) than women who birthed in a private facility (see Table 3).

**Table 3:** **Associations between sector of birth and postpartum care**

	**Public facility % (N)**	**Private facility** ^**1**^ ** % (N)**	**Adj OR (99%** **CI)** ^**2**^
**Length of hospital stay**			
*<24 hours*	8.2 (308)	0.2 (5)	5.60 (1.69-18.50)^***^
*1-2 nights*	43.6 (1635)	4.5 (121)	1
*3-4 nights*	35.0 (1313)	55.4 (1487)	0.07 (0.05-0.09)^***^
*5+ nights*	13.2 (495)	39.9 (1069)	0.03 (0.03-0.05)^***^
**Received a telephone call from a care provider**			
*No*	42.4 (1634)	82.7 (2218)	1
*Yes*	56.4 (2117)	17.3 (464)	6.28 (5.30 - 7.44)^***^
**Visited at home by a care provider**			
*No*	33.1 (1243)	93.6 (2510)	1
*Yes*	66.9 (2508)	6.4 (172)	34.23 (26.93 – 43.50)^**^
**Visited a nurse or midwife**			
*No*	80.7 (3072)	75.8 (2034)	1
*Yes*	19.3 (724)	24.2 (648)	0.72 (0.60 – 0.84)^***^
**Visited a GP**			
*No*	49.3 (1849)	83.6 (2243)	1
*Yes*	50.7 (1902)	16.4 (439)	5.57 (4.68 – 6.63)^***^
**Access to 24-hour support**			
*No*	16.5 (673)	19.5 (566)	1
*Yes*	83.5 (3397)	80.5 (2337)	1.30 (1.08 – 1.58)^***^

^**^p < .01, ^***^p < .001.

^1^Private sector is the referent category.

^2^adjusted for age, parity, level of education, country of birth, Aboriginal and/or Torres Strait Islander identification and area of residence.

### Factors associated with satisfaction with amount of postpartum care received

After adjustment for demographic differences, women who birthed in a public facility had twice the odds of being satisfied with the amount of postpartum care they received than women who birthed in a private facility (see Step 2, Table 4). However, the association between sector of birth and satisfaction with the amount of care was no longer evident after the inclusion of postpartum initiatives proactively delivered by health services (see Step 3, Table 4). After controlling for demographic factors, sector of birth, and all other types of postpartum care, women had significantly higher odds of being satisfied with their postpartum care if they had been telephoned or visited at home by a care provider within 10 days of being discharged, had access to the contact details of a care provider for 24-hour support and had visited a nurse/midwife or a GP (see Step 4, Table 4). Length of hospital stay was not associated with satisfaction with amount of postpartum care after accounting for all other factors (see Table 4).

**Table 4:** **Hierarchical multivariate logisitic regression to assess factors associated with satisfaction with the amount of postpartum contact** ^**1**^

	**Step 2** ^**2**^		**Step 3** ^**3**^		**Step 4** ^**4**^	
	**AOR**	**99%** **CI**	**AOR**	**99%** **CI**	**AOR**	**99%** **CI**
**Sector of birth facility**						
*Private facility*	1		1		1	
*Public facility*	2.10	1.76-2.52^***^	1.10	0.83-1.37	0.95	0.78-1.31
**Length of hospital stay**						
*<24 hours*	-		1.26	0.74-2.18	1.22	0.71-2.11
*1-2 nights*	-		1		1	
*3-4 nights*	-		0.98	0.78-1.28	1.04	0.81-1.33
*5+ nights*	-		1.05	0.79-1.39	1.07	0.80-1.43
**Telephoned by a care provider**						
*No*	-		1		1	
*Yes*	-		1.82	1.48-2.23^***^	1.79	1.46-2.19^***^
**Visited at home by a care provider**						
*No*	-		1		1	
*Yes*	-		2.26	1.79-2.86^***^	2.47	1.95-3.13^***^
**Access to contact for 24 hour support**						
*No*	-		1		1	
*Yes*	-		3.65	3.01-4.42^***^	3.64	3.00-4.42^***^
**Visited a nurse/midwife**						
*No*	-		-		1	
*Yes*	-		-		1.93	1.52-2.44^***^
**Visited a GP**						
*No*	-		-		1	
*Yes*	-		-		1.24	1.01-1.52^**^

**p < .01, ***p < .001.

^1^Step 1(not shown) simultaneously adjusted for age, parity, level of education, country of birth, Aboriginal and/or Torres Strait Islander identification, and area of residence.

^2^Step 2 adjusted for sector of birth.

^3^Step 3 adjusted for initiatives proactively delivered by health services (length of hospital stay, being telephoned by a care provider, visited at home by a care provider and being given contact details of someone to contact at any time).

^4^Step 4 adjusted for initiatives undertaken by women (visiting a midwife or nurse and visiting a GP).

### Factors associated with parenting confidence

Women who birthed in a public facility had 1.33 times the odds of being confident in their parenting than women who birthed in a private facility, after adjustment for demographic differences (see Step 2, Table 5). However, the significant association between sector of birth and parenting confidence was no longer evident after the inclusion of postpartum initiatives proactively delivered by health services (see Step 3, Table 5). After controlling for demographic factors, sector of birth, and all other types of postpartum care, women had significantly higher odds of parenting confidence if they had contact details for 24-hour support, and significantly lower odds of parenting confidence if they had visited a nurse or midwife in the 10 days after birth or stayed in hospital 3 nights or more (see Step 4, Table 5). Having received a telephone call or home visit, and having visited a GP, were not significantly associated with parenting confidence.

**Table 5:** **Heirachical multivariate logistic regression to assess factors associated with parenting confidence** ^**1**^

	**Step 2** ^**2**^		**Step 3** ^**3**^		**Step 4** ^**4**^	
	**AOR**	**99%** **CI**	**AOR**	**99%** **CI**	**AOR**	**99%** **CI**
**Sector of birth facility**						
*Private facility*	1		1		1	
*Public facility*	1.33	1.12-1.59^***^	1.17	0.92-1.47	1.16	0.91-1.48
**Length of hospital stay**						
*<24 hours*	-		1.20	0.85-1.69	1.21	0.86-1.71
*1-2 nights*	-		1		1	
*3-4 nights*	-		0.73	0.60-0.90^***^	0.73	0.59-0.89^***^
*5+ nights*	-		0.65	0.50-0.83^***^	0.64	0.50-0.83^***^
**Telephoned by a care provider**						
*No*	-		1		1	
*Yes*	-		1.04	0.88-1.23	1.04	0.88-1.24
**Visited at home by a care provider**						
*No*	-		1		1	
*Yes*	-		0.87	0.71-1.06	0.84	0.69-1.03
**Access to contact for 24 hour support**						
*No*	-		1		1	
*Yes*	-		1.33	1.07-1.64^***^	1.34	1.08-1.65^***^
**Visited a nurse/midwife**						
*No*	-		-		1	
*Yes*	-		-		0.80	0.66-0.98^**^
**Visited a GP**						
*No*	-		-		1	
*Yes*	-		-		1.01	0.86-1.20

**p < .01, ***p < .001.

^1^Step 1(not shown) simultaneously adjusted for age, parity, level of education, country of birth, Aboriginal and/or Torres Strait Islander identification, and area of residence.

^2^Step 2 adjusted for sector of birth.

^3^Step 3 adjusted for initiatives proactively delivered by health services (length of hospital stay, being telephoned by a care provider, visited at home by a care provider and being given contact details of someone to contact at any time).

^4^Step 4 adjusted for initiatives undertaken by women (visiting a midwife or nurse and visiting a GP).

### Factors associated with feeling depressed after birth

After controlling for demographic factors, the odds of feeling depressed sometimes or often for women who birthed in a private facility (32.2%) were not significantly different from women who birthed in a public facility (34.8%) (AOR =1.126, 99% CI = 0.97-1.13, *p* = 0.04; data not shown). Therefore, the potential mediating effects of postpartum care in associations between sector of birth and feeling depressed after birth were not further examined.

## Discussion

### Associations between sector of birth, postpartum care and satisfaction

This study found significant differences between sectors for the services received and provided for postpartum women following hospital discharge. Although women who birth in the public sector are more likely than women who birth in the private sector to be discharged from hospital less than 3 nights after birth,[24] they are also more likely to have contact with a health professional in the first 10 days after leaving hospital. This contact is either proactively organised by the health care system (e.g. telephone calls, home visits and provision of contact details) or self-initiated by women (e.g. by visiting a GP).

After adjusting for demographic variables including parity, highest level of education, maternal age, area of residence, country of birth and Aboriginal and/or Torres Strait Islander identification, women who birthed in a public facility had twice the odds of being satisfied with the amount of postpartum care than those who birthed in a private facility. However, the association between sector and satisfaction was mediated by postpartum care received. Women had higher odds of being satisfied if they had contact with a health professional within 10 days of hospital discharge (either organised by the hospital or self-initiated) or had been provided with the details of a person they could contact 24 hours a day regardless of where they birthed or their length of hospital stay.

Sector did not impact on whether women felt depressed sometimes or often after controlling for demographic factors. These findings are consistent with those found by Buist et al. [19] in which any effect of sector on depression was mitigated by education and income.

These results clearly demonstrate that women in the public sector are significantly more likely than women in the private sector to receive a telephone call or home visit and to visit their GP, regardless of length of stay, and that this contact has a corresponding positive association with maternal satisfaction with the amount of contact. From the public sector perspective, implementing UPNCS has ensured that the majority of women receive a telephone call and/or home visit within 10 days following discharge. Women who birth in the public sector in Queensland are also advised to visit their GP within the first two weeks postpartum. Compliance with this advice is reflected in the higher proportion of women birthing in the public sector accessing their GP. These strategies appear to be a positive step in improving maternal satisfaction with the amount of contact received.

In contrast, there are no comparable programs for early post-discharge contact for women who birth in the private sector in Queensland [30]. Home visiting has often been a feature of early discharge programs [34,35]. Women’s typically longer hospital stays in the private sector may explain why only a small proportion received a home visit. However, a longer hospital stay does not explain the lower incidence of telephone follow-up or self-initiated visits to a GP. A similar lack of availability of home visits by a domiciliary midwifery service for women in the private sector has also been noted in the States of Western Australia [20] and Victoria, [36] but there are no further data describing other health professional contacts in the postpartum period between sectors. Studies have found that even when women who birth in a private hospital are visited at home, they tend to be less satisfied with the style of midwifery care they received, particularly with regard to being given information that was easy to understand and having an active say in the treatment provided [20].

More broadly, the literature on the impact of universal postpartum contact is mixed, with some studies finding improvements in satisfaction [37], particularly with home visits [38-40], and others finding little difference in outcomes such as breastfeeding rates [41], depression, and hospital readmission [42-45]. While home visits were associated with higher odds of satisfaction with amount of postpartum contact in this study, other contacts were still effective. Therefore, it is important to assess the most cost effective method of providing postpartum care in the community so that maternal satisfaction and other outcomes are optimised.

### Associations between sector of birth, postpartum care and confidence

While women in the public sector had higher odds of being confident caring for their baby after hospital discharge, the association between sector and confidence did not persist when adjusted for postpartum health professional contact and length of stay. As mothers were asked to rate their confidence when first at home after their hospital stay, it is not surprising that confidence was not influenced by the amount of postpartum contact they received following discharge, from midwives, nurses or GPs. However, increasing length of hospital stay and visiting a nurse/midwife after discharge were associated with lower odds of being confident. These findings may be explained, in part, if the longer stay was due to a maternal or infant problem (including caesarean birth), and visits to a nurse/midwife were primarily because of difficulties experienced rather than a routine check. Nevertheless, it is unlikely that these factors can fully account for the differences found, especially as women birthing in private facilities routinely stay for more than 3 days. Regardless of length of stay, it is important that women feel empowered to care for their babies while in hospital. It may be the type of care received, rather than length of stay per se, that influences parenting confidence. Women commonly report receiving conflicting information when in hospital, particularly for breastfeeding [46,47] which may negatively impact parenting confidence. Although more than 80% of women from both sectors had the details of someone they could contact 24 hours a day, this was the only factor that improved a mother’s odds of being confident in her parenting ability.

In summary, differences in postpartum care are associated with differences in satisfaction with the amount of care between sectors, but not differences in confidence. While all forms of contact (telephone, home visit, or visiting a nurse/midwife or GP) were associated with improved odds of maternal satisfaction, there was not a similar improvement in the odds for confidence. Having access to a heath professional that women could contact 24 hours a day was associated with improved odds of both being satisfied and confident, irrespective of sector.

While it is often considered that women in the private sector receive better care, and are more satisfied with the quality of their in-hospital care [24,48], the lack of follow-up health professional contact is related to poorer levels of satisfaction with postpartum support [20,25]. Similarly, longer lengths of stay (3–4 nights), commonly found in the private sector, are thought to allow women time to recover from the birth [36] and provide an opportunity for mothers to become more confident managing their babies with professional support close at hand [25,49]. Some women view domiciliary midwifery visits as a poor substitute to in-hospital care, feeling more secure and having peace of mind while in hospital [25,49]. However, in this study, longer hospital stay was not associated with improved parenting confidence and the type of care women receive while in hospital may, in fact, disempower them in the early postpartum period.

### Limitations

The most important limitation of this study is the response rate of 35%. Confidential sampling via the Registry of Births, Deaths and Marriages meant we were unable to send tailored reminders to non-responders to encourage survey completion, or obtain further data determining the precise impact of the response rate on the representativeness of the sample. However, the marginal differences between this sample and the population of birthing women would be unlikely to affect the results of this study.

We have only included satisfaction with amount of postnatal contact, but there may be different associations between the type of care received or sought and satisfaction with the quality of care received postnatally. It is also possible that we have not accounted for all confounders. More research, particularly qualitative research, is needed to determine the relative effects of different types of contact (from who? for what?) on women’s perceptions of the quality of their care and other outcomes (breastfeeding, mental health, etc.).

## Conclusion

This study found that women birthing in the private sector had significantly lower odds of receiving health professional care after hospital discharge compared to women birthing in the public sector. To improve maternal satisfaction with post-discharge postpartum care women should routinely have contact with a health professional within 10 days of discharge, regardless of length of stay or sector of birth. This contact may be face-to-face or by telephone, and provider or mother initiated. Increased length of hospital stay does not compensate for lack of contact following discharge. Therefore, all women should have the expectation of speaking to and/or seeing a doctor, midwife or nurse soon after they are first at home with their baby.

Providing women with details of a person they can contact 24 hours a day if they have concerns will improve both satisfaction and confidence and is a simple and inexpensive first step to implement. Further research is needed to explore the factors that might explain the association between longer length of hospital stay and poorer confidence.

## Abbreviations

AOR: Adjusted Odds Ratio
CFHNs: Child and Family Health Nursing Services
GP: General practitioner
UPNCS: Universal Postnatal Contact Service
